# Compression-rate-dependent nonlinear mechanics of normal and impaired porcine knee joints

**DOI:** 10.1186/s12891-017-1805-9

**Published:** 2017-11-14

**Authors:** Marcel Leonardo Rodriguez, LePing Li

**Affiliations:** Department of Mechanical and Manufacturing Engineering, University of Calgary, 2500 University Drive, N.W, Calgary, AB T2N 1N4 Canada

**Keywords:** Articular cartilage, Compression-rate dependence, Knee joint mechanics, Mechanical test, Meniscus, Poromechanics

## Abstract

**Background:**

The knee joint performs mechanical functions with various loading and unloading processes. Past studies have focused on the kinematics and elastic response of the joint with less understanding of the rate-dependent load response associated with viscoelastic and poromechanical behaviors.

**Methods:**

Forty-five fresh porcine knee joints were used in the present study to determine the loading-rate-dependent force-compression relationship, creep and relaxation of normal, dehydrated and meniscectomized joints.

**Results:**

The mechanical tests of all normal intact joints showed similar strong compression-rate-dependent behavior: for a given compression-magnitude up to 1.2 mm, the reaction force varied 6 times over compression rates. While the static response was essentially linear, the nonlinear behavior was boosted with the increased compression rate to approach the asymptote or limit at approximately 2 mm/s. On the other hand, the joint stiffness varied approximately 3 times over different joints, when accounting for the maturity and breed of the animals. Both a loss of joint hydration and a total meniscectomy greatly compromised the load support in the joint, resulting in a reduction of load support as much as 60% from the corresponding intact joint. However, the former only weakened the transient load support, but the latter also greatly weakened the equilibrium load support. A total meniscectomy did not diminish the compression-rate-dependence of the joint though.

**Conclusions:**

These findings are consistent with the fluid-pressurization loading mechanism, which may have a significant implication in the joint mechanical function and cartilage mechanobiology.

## Background

The knowledge of joint mechanics is essential for understanding the mechanism of joint injury and disease. Applications of this knowledge also include joint reconstruction and replacement. Articular cartilage is essential for the normal mechanical function of a diarthrodial joint. The load response of articular cartilage is highly compression-rate dependent. For a given magnitude of compressive strain, the reaction force is substantially greater if the strain is applied at a higher rate until the force approaches its asymptote. This rate-dependent response is well observed from the mechanical tests of cartilage disks and elaborated in modeling [1–3]. The rate-dependence is also found in tensile tests of articular cartilage [4, 5]. However, these tests have been performed using small tissue explants. The rate-dependence has not been sufficiently investigated at the joint level to account for the complex structure of the joint and provide physiologically relevant data.

A series of experimental techniques have been used to study the mechanics of the knee joint. Hand-held probes are used during knee arthroscopy to evaluate cartilage properties [6–8]. Non-invasive methods, such as electroarthrography [9] and T2 relaxation maps of magnetic resonance imaging [10], are also used to determine the integrity of the tissue structure. These measurements target at cartilage properties rather than the mechanical response of the joint.

The mechanical testing using entire knee joint specimens is still the most direct and convenient measurement to gain the mechanical response of the joint [11–13]. Static assessment of cadaveric knee joints shows the role of the Medial Collateral Ligament (MCL) to primarily restrain valgus loading and stabilize external rotation [14–16]. Impact tests have been used to study the influence of braces on the MCL elongation for dynamic conditions, the multiple failure modes of the Posterior Cruciate Ligament (PCL) on a high-speed crash, and the increased anteromedial strain in the Anterior Cruciate Ligament (ACL) under combined impulsive knee compression, flexion and valgus moments [17–19].

The mechanical function of menisci has also been widely studied using joint testing [20–22]. A meniscectomized knee joint under compression shows a reduced contact area and increased peak pressure as compared with the intact joint, while any meniscal transplantation with only one horn secured gives intermediate values for both peak pressure and contact area [21–23]. Naturally, the contact area increases with force applied to the joint [24]. Cadaveric knee joint measurements showed that a significant fraction of the load is transmitted through the menisci [25]. In addition, a tibial flexion osteotomy increases cartilage pressure and causes an anterior shift in tibial resting position [26, 27].

Animal joints are widely used in the mechanical tests due to great availability, low cost and low biological risk as compared to the use of cadaveric joints. In addition, large animal stifle joints have a biological similarity to human knee joints [28, 29]. Porcine stifle joints are a popular choice for testing as the porcine knee has a similar size and anatomy to human’s [30, 31].

The mechanical response of the knee joint has been mostly studied for elastic or traumatic behavior [32–34]. Few studies have considered both transient and long-term mechanical behaviors [35]. The effect of loading rates on the joint response has only been addressed in the injury models of the joint. For example, two compression rates, 1 and 500 mm/s, were tested in a murine model of post-traumatic osteoarthritis [36]. Different injuries were seen when a compression of 1.7 mm was applied, respectively, at the two rates.

The investigation of rate-dependent mechanical behavior is essential for the understanding of the knee joint mechanics and the consequence of surgical procedures because the joint experiences a variety of loadings applied at different rates. The objective of the present study was to quantify experimentally the compressive loading-rate-dependent mechanical behavior of the knee joint and the subsequent creep and relaxation of the joint associated with the poromechanical behavior of the tissues in the joint. Only compressive loadings were considered in the study to highlight the influence of fluid pressurization in articular cartilages and menisci.

## Methods

Porcine stifle joints were used as they have anatomy similarities to human’s and are available in a large quantity required in the present study. Fresh porcine joints with sealed joint capsules were purchased within 24 h of the slaughter of the animals (Irricana Meat Market, Red Deer Lake Meat Processing and Ryan’s Meats, Alberta, Canada). The ankle joint was partially retained to keep the tibia and fibula together. The joints were kept in sealed bags with Phosphate Buffered Saline (PBS) and stored in a fridge at 4 °C before testing in 1–3 days.

**Fig. 1:**
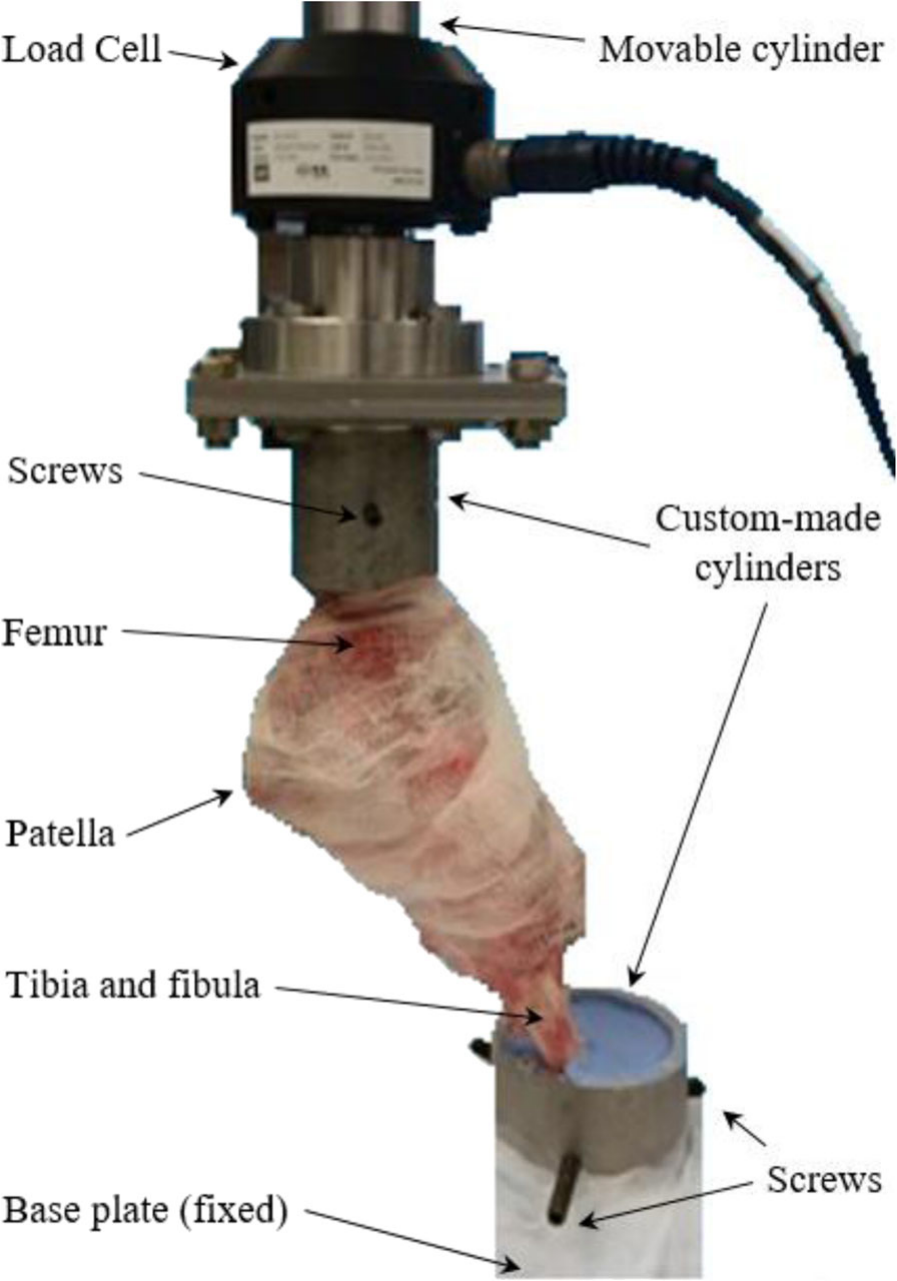
Experimental setup for the porcine knee joint test on MTS. The joint was initially fixed at its natural angle to custom-made adapters with screws and dental cement. The lower cylinder was fixed to the base of the MTS, while the upper one was able to move in the vertical direction only. A force was applied by the movable cylinder of the MTS

Compression tests of the joints were performed on an 858 Mini Bionix® II material testing system (MTS, Minnesota, USA). Two custom-made hollow steel cylinders, each welded at one end to a square flat plate, were used to fix the joint to the MTS (Fig. 1), as described below. All muscles were removed from the distal tibia and proximal femur so the ends of the specimen could be fixed in the hollow cylinders using dental cement (Fastray™ LC, Bosworth, IL, USA). Three alloy steel set screws were tightened to the bone surface before cementing, to prevent sliding between cement and cylinder or bone. The femur was fixed to the cylinder first in a fume hood, and the cement was let set for 10 min before the cylinder holding it was attached to a force transducer (MTS® 661.19F-02) using an adaptor. After the force transducer was installed in the movable cylinder of the MTS, the custom-made cylinder for the ankle joint was positioned appropriately on the MTS base to maintain the natural angle of the joint while the pig stands, which was approximately 40° [31]. The ankle joint was then fixed to the cylinder with screws and cement and finally let set for 15 min. This fixing procedure minimized malalignment and prestresses in the joint. Since the cementing of the ankle joint had to be done on the MTS, a fan was used to expel the cement fume to the fume hood using a long hose. PBS-soaked gauzes were used to cover the joint in order to keep it hydrated during the preparation and test (Fig. 1).

Station Manager®, a software developed by the MTS, was used to control the vertical motion of the load cell. Both force and displacement control modes are available. For safety reasons, the testing machine was set to stop if a compressive or tensile force reached 800 N, which did not actually occur during any tests. The sampling frequencies were set to 10–100 Hz, depending on the loading rate, for the loading phase, and 1 Hz for the relaxation/creep phase to record at least dozens of data points for each phase.

Before applying a targeted loading protocol, the joint was subjected to preconditioning with a cyclic loading, to ensure that the structure of the joint was at a repeatable reference state [37, 38]. After applying a compressive force of 20 N to ensure contact within the joint, the preconditioning was performed at 0.25 Hz with an amplitude of 400 μm for 30 cycles. The response tended to be repeatable in less than 10 cycles.

Measurements were performed for different loading protocols with intact, dehydrated, and meniscectomized joints. Six groups of mechanical tests were done with 45 joints in total (Table 1) as described below.

1. A pilot study (*n* = 13) was conducted to determine the appropriate magnitude of ramp compression within the elastic range and compression rates necessary to approximate the full range of rate-dependent response. Eleven joints were tested with an 800-μm ramp compression at six rates respectively, 10, 50, 100, 500, 1000 and 2000 μm/s, from the lowest to highest rate, followed by a 20-min relaxation period. Two additional joints were loaded with a higher ramp compression of 1200 μm at up to 5000 μm/s (Table 1). The joint remained unloaded for at least 20 min between tests on the same joint to allow tissue recovery from synovial fluid loss and deformation. The loading protocol was repeated for 2–3 times with the same specimen among some of these joints (7/13).
2. A ramp compression of 1200 μm with four compression rates, 10, 100, 1000 and 2000 μm/s, was chosen for most further tests after the pilot study. The relaxation phase was extended to 35 min for the increased compression of 1200 μm. The relaxation phase was skipped in some tests in order to complete multiple compression rates for the same specimen during the same day (Table 1).
3. Different loading sequences were tested with the same joint specimens, when various compression rates were applied, respectively, from the lowest to highest rate, from the highest to lowest rate and randomly starting from a middle rate (*n* = 5).
4. The static response was simulated with the equilibrium response of relaxation tests, because a zero compression rate cannot be possibly performed. A joint was slowly compressed (~10 μm/s) to 300, 600, 900 and 1200 μm, respectively, and each of the four compressions was followed by a relaxation of 30–45 min to static equilibrium (*n* = 7, Table 1).
5. The force-displacement relationship was examined with repeated tests on the same intact and dehydrated joints (*n* = 3). First, the fully hydrated intact joint was tested. Second, the joint capsule was carefully opened to drain the synovial fluid partially: dry kitchen paper towels were inserted above and below the menisci by lifting the femur slightly, followed by a small joint compression to squeeze out some fluid from the tissues. Finally, the towels were removed, and the same loading protocol was repeated on the joint after the fluid loss.
6. Tests were repeated on the same joints before and after meniscus removal (*n* = 12, Table 1). A ramp compression of either 1200 or 800 μm was applied in the displacement-control tests with or without the relaxation phase (*n* = 8). A force of 500 N was applied in 20 s and kept constant for 30–60 min in the creep loading protocol with additional specimens (*n* = 4). After the test of each intact joint, small incisions were made to reach and cut the meniscal attachments and remove the menisci from the joint, while the specimen remained on the MTS. The joint was then tested with the same loading protocol. The locations of the incisions were chosen to keep the synovial fluid in the joint capsule.

**Table 1:** Summary of six groups of mechanical tests performed for specific purposes

Tests	Intact					Dehydration	Meniscectomy		
Purpose of Tests	(1) Pilot study		(2) Rate dependence	(3) Altered test sequence	(4) Static response	(5) Fluid pressure support	(6) Load support of menisci		
Applied peak force/displacement	800 μm	1200 μm	1200 μm			1200 μm	1200 μm	800 μm	500 N
Rates in loading phase	10, 50, 100, 500, 1000, 2000 μm/s	10, 100, 1000, 2000, 5000 μm/s	10, 100, 1000, 2000 μm/s	10, 100, 1000, 2000 μm/s	< 10 μm/s	10, 100, 1000, 2000 μm/s	10, 100, 1000 μm/s	10, 100, 1000 μm/s	25 N/s
Minutes for relaxation/creep phase	20	0 (no relaxation)	35 (3 joints); 0 (2 joints)	0	30–45	0	30–60	30–60 (3 joints); 0 (3 joints)	30–60
Joint angle	~40°	~40°	~40°	~40°	~40°	~40°	~40°	30°	30°
Number of joints	11	2	5	5	7	3	2	6	4

The total number of joints was 45. The tests of dehydrated and meniscectomized joints were performed after the mechanical tests of the corresponding intact joints

All tests were performed when the joints were held at their natural angles (~40°) with a 10-kN force transducer until the shared load cell failed (*n* = 35; Table 1). Although the failure of the load cell was unlikely related to the loading tests in this study, we reduced the joint angle to 30° to reduce the horizontal reaction and bending moment in the system, which were potential risks for the force transducer. We further reduced the maximum ramp compression from 1200 back to 800 μm in the sixth group of tests (Table 1). A new force transducer of 15-kN was used thereafter (*n* = 10). The resolution is 1% in force for this type of transducers according to the manufacturer.

The joints were carefully opened and visually inspected for tissue damage and degeneration after the mechanical tests. Only two joints from all tests were found to be abnormal: one had a small cut in the joint capsule resulting in a synovial fluid loss, another had a frozen joint capsule because the lab refrigerator was accidentally set to an unusually low temperature. The one with a damaged joint capsule showed a much smaller reaction force than other joints. The one with a frozen joint capsule showed a much weaker rate-dependence in the load response. Therefore, the measurements from these two joints were excluded from Table 1.

Finally, all statistical analyses were performed with Minitab 17.1.0 (Minitab Inc., PA, USA). The General Linear Model in the software includes a two-way ANOVA (analysis of variance) that was used, for example, to determine the significance of our results on the compression-magnitude, compression-rate and the combined effect of the two variables.

## Results

The force-displacement data for various rates were reproducible when the same loading protocol was repeated on the same joints (*n* = 7 which includes all joints in Group 1 that were subjected to repeated tests). For example, the relative error was 4.0% at 800 μm, and increased to 7.4% at 400 μm compression when averaged over all compression-rates for all repeated tests. No significant difference was found between repeated tests as shown in a two-way ANOVA (*p* = 0.93). The deviations produced by different loading sequences were also insignificant (two-way ANOVA *p* = 0.88; *n* = 5, Group 3). The relative error was 2.2% and 6.3%, respectively, at 1200 μm and 600 μm compression. These findings validated the testing system including the fixing procedure. They also indicated that the loadings were within the elastic limit of the joints and that the waiting periods between tests on the same joints were sufficient for the tissue recovery from the previous loading.

**Fig. 2:**
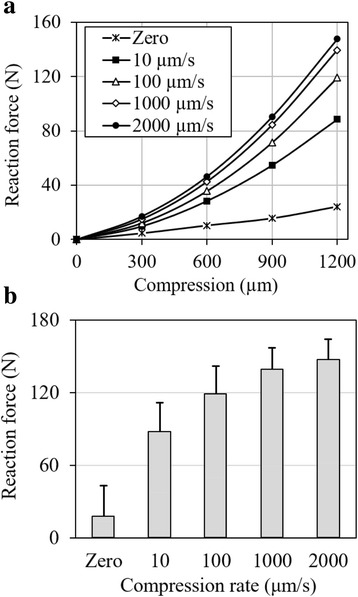
Average reaction force in the joint as a function of (**a**) compression magnitude and (**b**) compression rate (*n* = 6). The data for the zero compression rate were taken from the equilibrium responses of the relaxation tests of six joints when each joint was compressed, respectively, by 300, 600, 900 and 1200 μm. **b** shows the reaction for the 1200-μm compression: the average forces were 24, 88, 119, 139 and 148 N, when the equal compression of 1200 μm was applied, respectively, at a compression rate of 0, 10, 100, 1000 and 2000 μm/s (specimens were obtained from the same source)

The average force-compression relationship is shown for five compression rates (Fig. 2a). For the clarity of the figure, the deviations are shown separately for 1200-μm compression only (Fig. 2b), which also shows the force as a function of compression rate. A two-way ANOVA showed a significant dependence of the reaction force on the compression-magnitude, compression-rate and the interaction between the two factors (*p* < 0.001 for all).

**Fig. 3:**
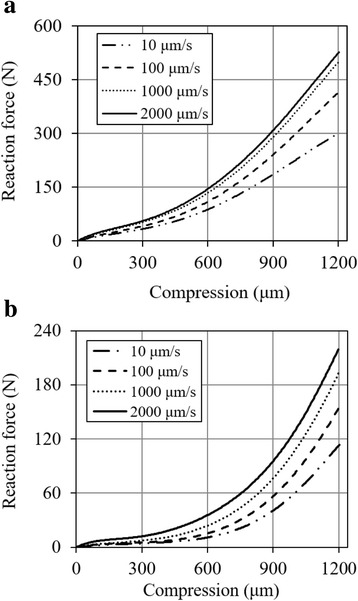
Influence of joint hydration on the compression-rate-dependent response of a knee joint: **a** intact joint capsule; **b** drained joint capsule. Note that the vertical axis of (**b**) is scaled to 40% of that of (**a**)

The reaction force was reduced by approximately 60% for all four compression-rates even when the synovial fluid was only partially removed (Fig. 3). An ANOVA analysis showed a significant dependence of the reaction force on the joint hydration (p < 0.001). Moreover, after the fluid loss from the tissues, the toe region of the force-displacement curve became more obvious and the force continued to increase substantially from 1000 to 2000 mm/s (Fig. 3b).

**Fig. 4:**
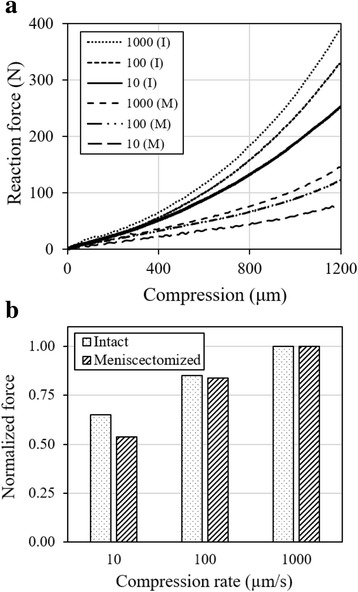
Influence of meniscectomy on the load support of the joint. The joint was first tested with intact joint capsule (I) and then after meniscectomy (M). **a** Typical reaction force as a function of compression for three compression rates, 10, 100 and 1000 μm/s respectively; **b** Normalized reaction force at 1200-μm compression: the force was normalized by the maximum obtained, respectively, from the intact and meniscectomized joint

**Fig. 5:**
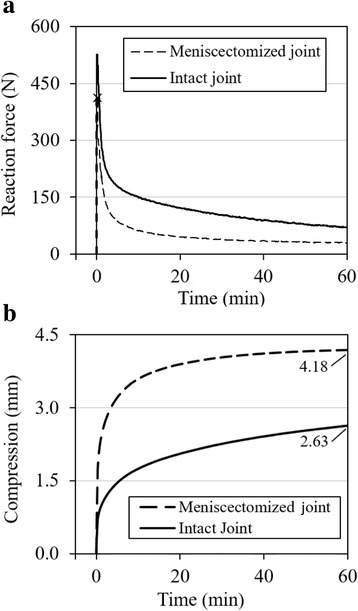
Typical transient responses of a knee joint before and after meniscectomy. **a** Relaxation test: the joints were compressed by 800 μm at a rate of 100 μm/s prior to relaxation. The peak forces were 419.8 ± 129.1 and 187.4 ± 112.3 N (n = 6), respectively, before and after meniscal removal of the same joints (× marks the peak force for the meniscectomized joint). **b** Creep test: the joints were loaded to 500 N in 20 s prior to creep. The maximum knee compressions were 3.24 ± 0.70 and 4.56 ± 0.98 mm (*n* = 4), respectively, before and after the meniscal removal

The reaction force for any compression rate was significantly reduced after meniscectomy (p < 0.001). At the compression of 1200 μm, the force was reduced by over 60% with meniscectomy (Fig. 4a). On the other hand, a larger percent variation in the rate-dependence was observed after meniscectomy (Fig. 4b). The long-term response was also altered by meniscectomy (Fig. 5). After the initial ramp compression, reaction force decayed rapidly within the first five minutes relaxation (Fig. 5a). The knee compression at equilibrium was increased by almost 40% with meniscectomy when a 500-N force was applied (Fig. 5b).

**Fig. 6:**
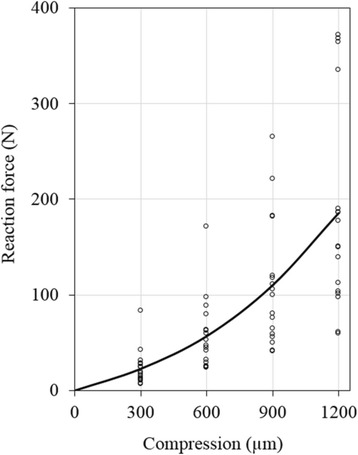
Force-compression data variation potentially caused by different ages and breeds of the pigs. The curve shows the average over the 17 intact joints, including 2, 5, 5, 3 and 2, respectively, from test Groups 1, 2, 3, 5 and 6 (Table 1). Data were taken from all joints (regardless of the sources) tested at the natural angle (~40°) with a compression of 1200 μm applied at 1000 μm/s

**Fig. 7:**
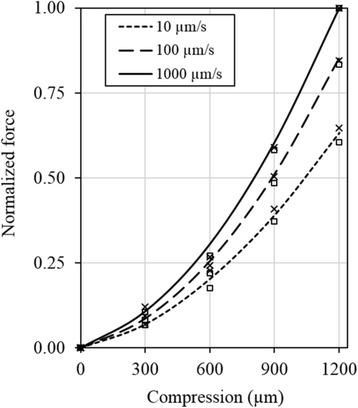
Normalized reaction force at three compression rates for the normal intact joints. The lines show the average results taken from Fig. 2a, while the symbols show the results for individual joints (□: Figs. 3a; ×: 4a). The value shown was normalized by the reaction force at 1200-μm compression applied at 1000 μm/s in each case

The reaction forces varied approximately 3 folds among different groups of joints (Fig. 6), which was most likely related to the age and breed of the animals that were not made available for this study. For this reason, only the joints obtained from the same source were used to evaluate the mean and deviation values (Fig. 2). Interestingly, the percent variations for different intact joints were similar as a function of either compression-magnitude or compression-rate (Fig. 7).

## Discussion

Forty-five porcine stifle joints were tested to determine the compression-rate-dependent force-compression relationship, creep or relaxation behavior of the normal, dehydrated, and meniscectomized knee joints. The joints were loaded with a flexion angle but only the vertical motion of the femur was possible. The testing system and loading protocols were reasonably examined with repeatable results.

The force-compression relationship of normal joints was predominantly determined by the compression rate (Figs. 2, 3a, 4 and 7). For a given magnitude of knee compression, the force increased with the compression rate rapidly until after 100 μm/s; it then increased slowly towards its asymptote (Fig. 2b); little increase was seen after 2000 μm/s, as indicated in the pilot study with a compression rate of 5000 μm/s (Table 1). In other words, a full-range of compression-rate-dependence has been approximated in the present study. On the other hand, the nonlinear behavior was also rate-dependent: the load response was almost linear at a nearly static compression; it became strongly nonlinear at a fast compression.

A comparison of these results with that from the literature is only partially available. Compression test data from cartilage discs showed over 10 times variation in stress over strain-rate for a given strain-magnitude of 10% [1]. Similarly, the dynamic modulus of cartilage tested in unconfined compression at 10 Hz cyclic loading was 24 times equilibrium modulus [39]. The present study indicated a weaker compression-rate dependence at the joint level than at the tissue level, likely due to non-uniform tissue compression and meniscus load support in the joint, as compared to uniform compression in the simple tissue test. Interestingly, the present study showed a variation of stress as a function of strain-rate that agrees with a model prediction. The model predicted a faster increase in the stress when the strain-rate gradually increases until around the vicinity of 5%/s then a slower increase towards to the asymptote [40]. The present study showed a similar trend (Fig. 2, 100 μm/s likely corresponds to ~5%/s average strain-rate in the joint), where the reaction force was only increased by 24% (119 to 148 N) when the compression-rate increased from 100 to 2000 μm/s (Fig. 2b). This result also compares well with the result from unconfined compression tests where the average dynamic modulus was only increased by 34% (48.2 to 64.8 MPa) when the frequency increased from 0.1 to 40 Hz [41]. Note that the present study only concerned with elastic deformation, as the variation of stress with strain-rate may be reversed at the strain level that causes the interruption of the collagen network [42].

The tissue hydration played a key role in the load support of the knee joint as indicated by the reaction force for a given joint compression. Fluid loss in the tissues not only reduced the load support of the joint, but also altered its nonlinear mechanical behavior including a more obvious toe region and a delayed asymptote for the compression rate (Fig. 3b vs 3a). This observation may be explained by fluid pressurization in the issues: some partially saturated region became fully saturated and pressurized with tissue compression. Therefore, the force increased faster with further compression. The fluid-pressure load support in the tissue and joint was explored previously [43, 44]. Therefore, only three joints were used in the present study (Table 1) to demonstrate the phenomenon and the compromised load support was not quantitatively correlated to the amount of fluid loss in the joint. On the other hand, it is understood from the literature that a partial fluid loss does not affect the equilibrium response of the joint because tissue hydration has little influence on the response at low compression rates [45, 46] when the load is mainly supported by the tissue matrix [47, 48]. Thus, the dehydration tests were only performed for the loading phase without creep or relaxation phases.

The load support of the joint was compromised consistently over all compression rates after a total meniscectomy. For the compression of 1200 μm, the total meniscectomy reduced approximately 60% of the load support as compared to that of the same intact joint at the same compression rate (Fig. 4a). The reduction was slightly greater at lower compression rates (68% and 62% reductions at 10 and 1000 μm/s respectively). The menisci were known to improve the congruency in the knee and thus provide a better load support in the joint [49–52]. Our result compares well with the previous data from partially degenerate human and healthy pig knees that showed menisci bearing 45–75% of the joint load [53]. Interestingly, the patterns of rate-dependence for normal and meniscectomized joints were similar (Fig. 4b). The slightly larger variation in the meniscectomized joint was probably caused by a greater contact area change during loading as compared to the normal joint. Furthermore, a meniscectomy also altered the rate of creep and relaxation and greatly reduced the load support of the knee at equilibrium (Fig. 5) while the fluid loss does not affect the equilibrium response. This observation was supported by modeling results that showed changes in fluid pressurization after both total and partial meniscectomy [54, 55].

The mechanical responses of all normal joints were found to have similar patterns of compression-rate dependence (Fig. 7), although the stiffness of the joint in compression varied over 3 times among the joints (Fig. 6). This implied that the rate-dependent response was governed by the same mechanisms regardless of differences in the age or size of the joints tested in the study. The load response was mainly determined by cartilages and menisci in the present test conditions. It has been understood from constitutive modeling that the interplay between fibril-reinforcement and fluid pressurization governs the mechanical behavior of articular cartilage in unconfined compression [3, 56]. The necessity of implementing this fluid pressurization mechanism in cartilages and menisci was also demonstrated with a human knee joint model [57]. The mechanism was partially validated in the present study when the fluid loss in cartilages and menisci greatly reduced the load support of the joint (Fig. 3).

There were a few limitations with the present study. First, the age and breed of the animals were not available, because all the pigs were butchered for meat. Second, only one flexion angle was tested for each joint due to the constraint of the adapters; the corresponding loading condition is similar to that of a standing animal. Third, the system could only record the vertical reaction force and thus the knee response to the transverse loading and bending was not investigated. Finally, the horizontal force and bending moment in the joint required the use of large load cells (10-kN and 15-kN), although the vertical force was well below 1 kN. As a result, we were not able to obtain good data for a very slow compression at 1 μm/s (the data for static compression were taken from the equilibrium response of relaxation tests). However, these limitations do not compromise the qualitative results of the present study.

## Conclusions

The force-compression relationship of the fresh porcine joint is highly compression-rate-dependent, which is demonstrated in the present study to be greatly influenced by the fluid pressurization in the cartilaginous tissues. This phenomenon has been previously observed in the mechanical testing with cartilage explants that are immersed in PBS. The present results have been obtained using the whole joints with sealed joint capsules and thus the cartilaginous tissues are intact, saturated with only synovial fluid and subjected to realistic contact and loading conditions. Accordingly, a weaker rate-dependence has been observed in the joint than explant tests, while the trend of variation on the strain-rate is similar.

This study has also confirmed the role of menisci in the creep and relaxation behavior of the joint, in addition to the previous finding in the load share in the joint.

Although no animal models will ultimately match human joints, we expect similar mechanical behaviors for the human knee joints, as predicted by our computer simulations of the human joint. Since our daily life involves loading and unloading in the joint at different rates, the results presented here may help understand the injury, repair and mechanobiology of the knee joint.

## Abbreviations

ACL: Anterior cruciate ligament
ANOVA: Analysis of variance
MCL: Medial collateral ligament
MTS: Material testing system
PBS: Phosphate buffered saline
PCL: Posterior cruciate ligament
